# Comprehensive Analysis of TRP Channel-Related Genes for Estimating the Immune Microenvironment, Prognosis, and Therapeutic Effect in Patients With Esophageal Squamous Cell Carcinoma

**DOI:** 10.3389/fcell.2022.820870

**Published:** 2022-03-04

**Authors:** Fangchao Zhao, Shaolin Gao, Xuebo Qin, Ren Niu, Zhirong Li, Chuan Wang, Shujun Li

**Affiliations:** ^1^ Department of Thoracic Surgery, The Second Hospital of Hebei Medical University, Shijiazhuang, China; ^2^ Department of Thoracic Surgery, Hebei Chest Hospital, Shijiazhuang, China; ^3^ Department of Oncology, The Second Hospital of Hebei Medical University, Shijiazhuang, China; ^4^ Clinical Laboratory Center, The Second Hospital of Hebei Medical University, Shijiazhuang, China; ^5^ The Second Hospital of Hebei Medical University, Shijiazhuang, China

**Keywords:** esophageal squamous cell carcinoma, TRP channel-related genes, prognostic signature, immune microenvironment, TCGA

## Abstract

The Nobel Prize in Physiology or Medicine for the year 2021 was awarded to Ardem Patapoutian and David Julius for their discoveries of temperature-sensitive receptors (TRP channels) and tactile receptors (Piezo channels), both of which were previously unknown. TRP channels are at the heart of the human ability to detect temperature, and they also play crucial regulatory functions in the occurrence and progression of cancer. Despite this, there have been no research conducted on the prognostic significance of TRP channels in individuals with esophageal squamous cell carcinoma (ESCC). In GEO and TCGA cohorts, unsupervised clustering was first conducted based on 18 TRP channel-associated differentially expressed genes (DEGs) extracted from MSigDB database and KEGG database. Two TRP subtypes were identified and patients in subtype B had the best prognosis among the two subtypes. Significant differences in staging and grading existed among the different subtypes. In GEO cohort, univariate Cox analysis were performed to screen prognosis related genes. A TRP channel-related prognostic signature, which included 7 signature-related genes, was constructed by the least absolute shrinkage and selection operator (LASSO) Cox regression. Patients were divided into a high-risk group and low-risk group by the median risk score. In GEO and TCGA cohorts, Receiver operating characteristic (ROC) curves, principal component analysis (PCA), and univariate and multivariate Cox regression were performed to confirm the validity of signature. Following a more in-depth study of the TME based on the risk signature, it was discovered that the high-risk group had higher immune cell infiltration and lower tumor purity, indicating a bad prognosis. Patients with high risk scores also had increased immune checkpoint expression, indicating that these patients may be more likely to benefit from immunotherapy than other patients. We also found that paclitaxel, cisplatin, and 5-fluorouracil displayed a better response in treating the low-risk score ESCC patients. This study also adopted GTEx and qRT-PCR to perform experimental verification processes. In summary, we identified a TRP channel-associated prognostic signature. This signature can predict prognosis and immune microenvironment in ESCC.

## Introduction

Esophageal cancer is the fifth most prevalent malignancy in China and the fourth leading cause of cancer-related fatalities (Anandavadivelan and Lagergren, 2016). Esophageal cancer may be classified into two subgroups based on its histological characteristics: esophageal adenocarcinoma (EAC) and esophageal squamous cell carcinoma (ESCC) (Smyth et al., 2017). ESCC accounts for nearly 90 percent of all esophageal cancer occurrences in China and is the most frequent histologic form of the disease (He et al., 2020). Despite the breakthroughs in the diagnosis, prognosis, and treatment of ESCC, early diagnosis still remains poor, with a 5-year overall survival rate less than 20% in some cases (Yang et al., 2020). The occurrence and development of ESCC relies on multiple factors, stages, and genes. Accordingly, both genetic and environmental factors affect the development of this disease (Abnet et al., 2018). Increasing our understanding of the molecular mechanisms underlying the development of ESCC and improving the efficiency of screening clinical indicators are crucial for early diagnosis and improved prognosis in patients with ESCC.

Transient receptor potential (TRP) channels is a non-selective cation channel superfamily, and primarily involved in calcium homeostasis and calcium signaling (Nilius and Owsianik, 2011; Kaneko and Szallasi, 2014). The survival, propagation, aggressiveness, and treatment resistance of tumor cells are all influenced by calcium-dependent mechanisms. As a result, TRP channels have been proposed as potential powerful modulators of carcinogenesis and progression (Ouadid-Ahidouch et al., 2013; Pan et al., 2022; Rodrigues et al., 2016). According to various studies, the expression and/or activity of the TRP channels was altered in cancers (Anderson et al., 2019). The presence of the TRPV, TRPM, and TRPC subfamilies, in particular, has been shown to be related with the development and progression of cancer (Prevarskaya et al., 2007). Kudou et al. (2019) found that TRPV2 regulates cancer progression by affecting WNT/β-catenin or basal cell carcinoma signaling, and that TRPV2 strong expression is associated with a worse prognosis in ESCC patients. In ESCC cells, TRPC6 can arrest the cell cycle in G2/M phase by inhibit elevation of [Ca^2+^] and activation of Cdc2 kinase, thereby inducing cell apoptosis (Zhang et al., 2013; Zhang et al., 2017). Lan et al. (2019) proved that TRPM8 regulated PD-L1 expression by calcineurin-NFATc3 pathway in esophageal cancer cells. However, there is still a lack of bioinformatic research on TRP channel-related genes in ESCC.

The current research observed that the established clustering subtypes and TRP channel-associated prognostic signature are singificant for enhancing clinical risk stratification to enable administration decision-making and forecast prognosis for patients with ESCC. Besides, the prognostic value, effect on the tumor microenvironment (TME), response to immune checkpoints, and drug sensitivity of the TRP channel-associated genes in ESCC patients were explored completely on basis of the prognosis signature for the further effect determination of TRP channels on the TME. Moreover, the expression profiles of key genes were detected in clinical tissue samples.

## Materials and Methods

### Dataset Collection

RNA sequencing data and clinical information of ESCC patients were obtained from the TCGA database (80 tumor samples and 11 normal samples) and the GEO database (GSE35624, including 119 tumor samples). Meanwhile, the copy number variation (CNV), single nucleotide variation (SNV) and methylation data of ESCC were downloaded from TCGA database. To collect TRP channel-related genes, we downloaded the Reactome_TRP_channels gene set from the MSigDB database, and another gene set from the KEGG database: inflammatory mediator regulation of TRP channels. Finally, we obtained 120 TRP channel-related genes for subsequent analyses.

### Mutation Status and Differential Expression Analysis

Differential expression of TRP channel-related genes in tumor and normal tissues was detected using the “limma” R package. The “maftools” package was used to generate a waterfall plot of the mutation frequency of differentially expressed genes (DEGs) in ESCC patients. A protein-protein interaction (PPI) network was mapped with online tools on the GeneMANIA website. In addition, the gene set variation analysis (GSVA) of R software (Hänzelmann et al., 2013) was employed to predict the activation or inhibition status of TRP channel-related DEGs and cancer-associated pathways.

### Calculation of Transient Receptor Potential Risk Score

Two cohorts were used to validate the prediction performance while one of the cohorts (GEO-ESCC, GSE35624) was used to construct the prognostic signature. Significant prognostic genes were identified by univariate Cox regression analysis (*p* < 0.1). Subsequently, we performed the least absolute shrinkage and selection operator (LASSO) Cox regression analysis and multivariate Cox regression analysis using the “glmnet” package to screen for genes involved in the TRP scoring formula. The TRP risk score was calculated as: (gene 1 × expression coefficient) + (gene 2 × expression coefficient) + … + (gene n × expression coefficient). All patients in TCGA and GEO were divided into two groups (low-risk group and high-risk groups) according to the median value of TRP risk score. The overall survival (OS) between the high-risk and low-risk groups was compared with Kaplan-Meier analysis. In addition, principal component analysis (PCA) analysis was used to determine the ability of the TRP risk score to discriminate between patients. The receiver operating characteristic (ROC) analysis was performed using the “timeROC” R package. Cox regression, both univariate and multivariate, were used to identify independent prognostic factors in patients with ESCC. The nomogram was created and their performance was evaluated using the “rms” R package.

### Immune Status and Drug Sensitivity Analysis

The CIBERSORT algorithm calculates the proportion of different immune cell types based on the expression levels of immune cell-related genes. The output of the 22 infiltrated immune cells was integrated to generate an immune cell matrix for subsequent analysis. We performed Gene set enrichment analyses (GSEA) to identify 672 GO terms and 30 KEGG pathways associated with different risk groups. In addition, we used the “pRRophetic” package to predict the sensitivity of different risk groups to chemotherapy.

### Tissue Samples and Quantitative Real-Time Polymerase Chain Reaction

A total of 10 tumor tissue samples and nearby normal esophageal tissue samples were obtained from ESCC patients who underwent tumor resection. All tissue samples were collected from the Thoracic Surgery Department of the Second Hospital of Hebei Medical University with the approval by the Medical Ethics Committee of the hospital. In liquid nitrogen, fresh tumor and non-tumor tissues were frozen in a snap, preserving their integrity. The extraction of RNA was carried out using the TRIzol Reagent (Invitrogen, United States). PrimeScriptTM RT reagent Kit with gDNA Eraser (Takara) was adopted to synthesize complementary DNA (cDNA) by reverse transcription reaction. The SYBR Premix Ex Taq (Takara) was adopted for the qRT-PCR analysis. The normalization of all expression data to GAPDH as an internal control was made with the 2^−ΔΔCT^ approach. Sangon Biotech (Sangon Biotech, Shanghai, China) was applied to chemically synthesize all primers. Supplementary File S1 lists the primer sequences and original data. The score was further standardized and simplified to generate a riskscore. The score was subsequently mapped by subtracting the minimum and dividing by the maximum. Mapping was conducted to facilitate the interpretation of results from different platforms. The riskscore was calculated as follows: Riskscore (qRT-PCR) =(Score-Min)/Max.

### Statistical Analysis

The R software (v.4.0.1) was employed to perform all statistical analyses. The bioinformatics approach section covers detailed statistical approaches about Transcriptome information. *p* < 0.05 was of statistical significance.

## Results

### Expression Profile of Transient Receptor Potential Channel-Related Genes in Esophageal Squamous Cell Carcinoma

**FIGURE 1:**
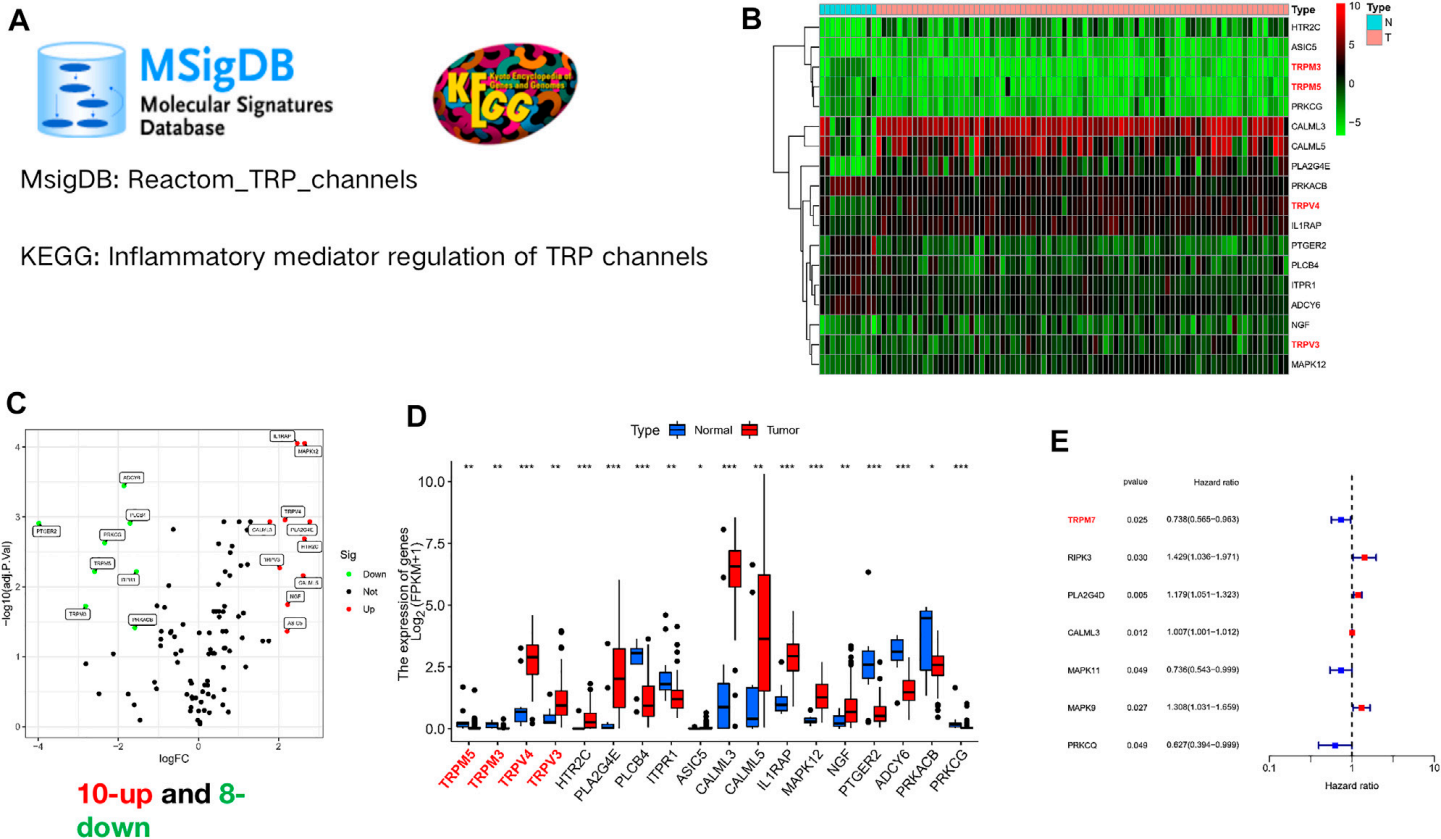
Expression profile of TRP channel-related genes in ESCC. **(A)** The original gene sets of TRP channels. A gene set (Reactom_TRP_channels) from the MSigDB database, and another gene set (inflammatory mediator regulation of TRP channels) from the KEGG database. **(B)** Heat map of TRP channel-related genes expression. The genes marked in red are members of the TRP superfamily, including TRPM3, TRPM35, TRPV4, and TRPV3. **(C)** The volcano plot of TRP channel-related differentially genes expression. Black dot, blue dot and red dot stand for no-statistical significance genes, low-expression genes and high expression genes, respectively. **(D)** The boxplot displayed the diversity in expression levels of 18 TRP channel-associated DEGs in tumor and normal tissues. **(E)** The forest plot showed the outcomes of univariate Cox regression analysis of TRP channel-associated genes. *p < 0.05, ***p < 0.001, ****p < 0.0001, ns, not significant.

The flow chart of this study is shown in Supplementary Figure S1. We obtained a gene set (Reactom_TRP_channels) from the MSigDB database, and another gene set (inflammatory mediator regulation of TRP channels) from the KEGG database, as shown in Figure 1A. Finally, 120 TRP channel-related genes were collected for subsequent analyses. RNA-seq data from the TCGA-ESCC cohort were analyzed using the limma package (*p* < 0.05; |logFC| > 1.5), and a total of 18 TRP channel-associated DEGs were identified in 80 ESCC and 11 normal samples (Figure 1B). In the heat map, the genes marked in red were members of the TRP superfamily, including TRPM3, TRPM35, TRPV4, and TRPV3. In addition, the volcano map showed that 10 genes were upregulated and 8 genes were downregulated in tumor samples compared to normal samples (Figure 1C). Specifically, the 10 upregulated genes were: TRPV4, TRPV3, HTR2C, PLA2G4E, ASIC5, CALML3, CALML5, IL1RAP, MAPK12, and NGF. 8 downregulated genes were TRPM5, TRPM3, PLCB4, ITPR1, PTGER2, ADCY6, PRKACB, and PRKCG (Figure 1D). In addition, to further verify the prognostic capacity of TRP channel-related genes, univariate Cox regression analysis was employed for screening (*p* < 0.05). TRPM7, MAPK11, and PRKCQ were identified as “protective” factors for ESCC patients, while PLA2G4D, RIPK3, CALML3 and MAPK9 were identified as “risk” factors (Figure 1E). Of note, in the TRP superfamily, only TRPM7 has a prognostic value in ESCC.

### Different Transient Receptor Potential Molecular Subtypes in Esophageal Squamous Cell Carcinoma Patients

**FIGURE 2:**
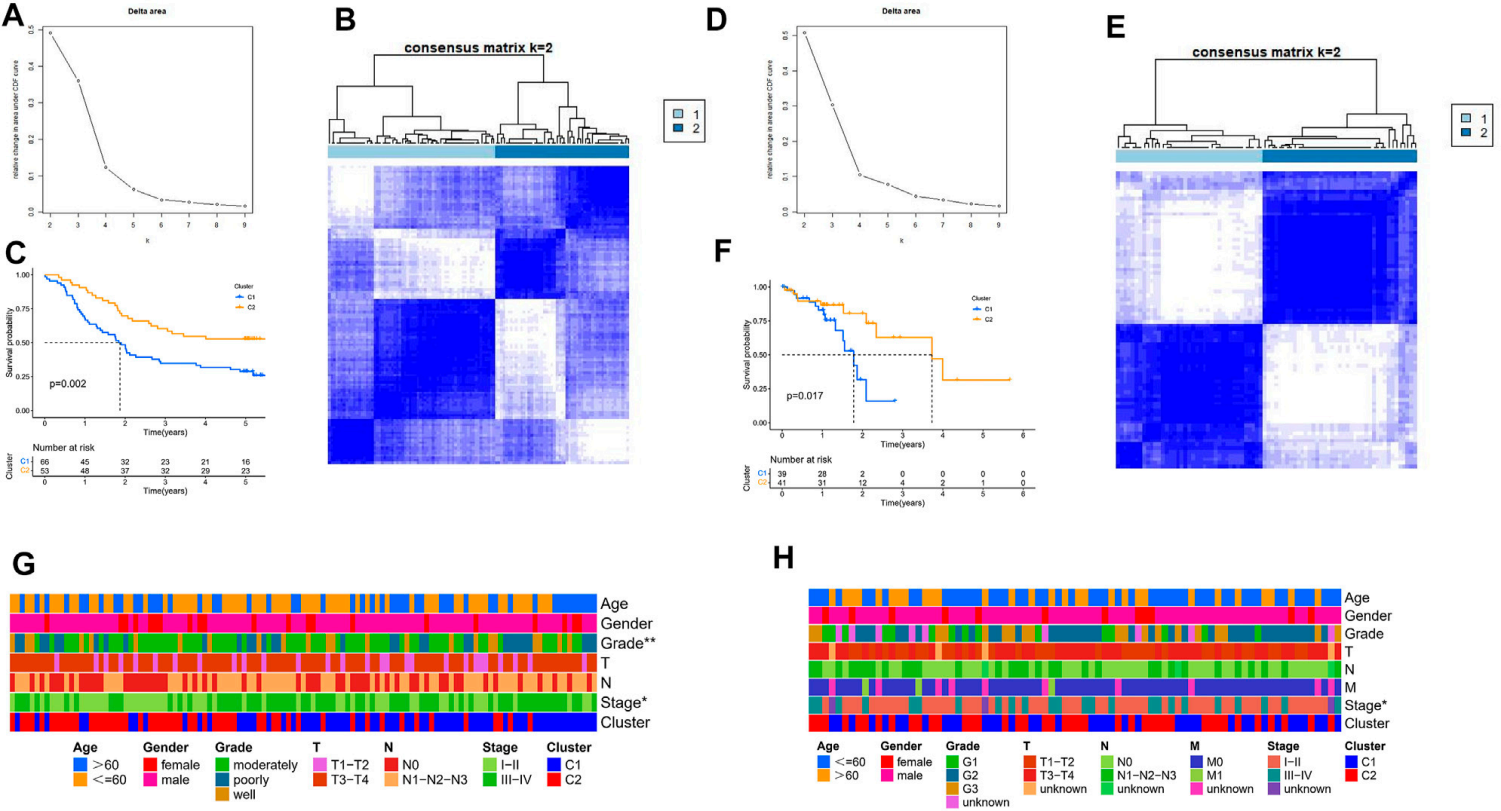
Consensus clustering of TRP channel-related DEGs. **(A)** Relative variation in region under CDF curve for k = 2 to 9 in the GEO cohort based on different expression genes. **(B)** The ESCC cohort from GEO was fallen into two different clusters when k = 2. **(C)** Kaplan-Meier survival analysis in different clusters in the GEO cohort. **(D)** Relative variation in region under CDF curve for k = 2 to 9 in the TCGA cohort based on different expression genes. **(E)** The ESCC cohort from TCGA was fallen into two different clusters when k = 2. **(F)** Kaplan-Meier survival analysis in different clusters in the TCGA cohort. **(G)** Comparison of the association between the clinicopathological features of two clusters in the GEO cohort. **(H)** Comparison of the association between the clinicopathological features of two clusters in the TCGA cohort.

To explore new subtypes of TRP-related molecules in ESCC patients, we performed a consensus clustering analysis. In the GEO-ESCC cohort, the highest intra-group correlations and lowest inter-group correlations were observed when k = 2, indicating that patients could be divided into two clusters based on 18 TRP channel-associated DEGs (Figures 2A,B). Similarly, the TCGA-ESCC cohort was also divided into two clusters (Figures 2D,E). Survival analysis showed statistically significant differences between different molecular subgroups (*p* < 0.05, Figures 2C,F). There were statistically significant differences in staging and grading between subtypes in the GEO-ESCC cohort (Figure 2G). Staging between different subtypes in the TCGA-ESCC cohort also showed significant differences (Figure 2H). In summary, we have argued for the significance of TRP channel-related genes on survival and tumor progression in ESCC patients from another perspective.

### Mutation Analysis of Transient Receptor Potential Channel-Related Genes

**FIGURE 3:**
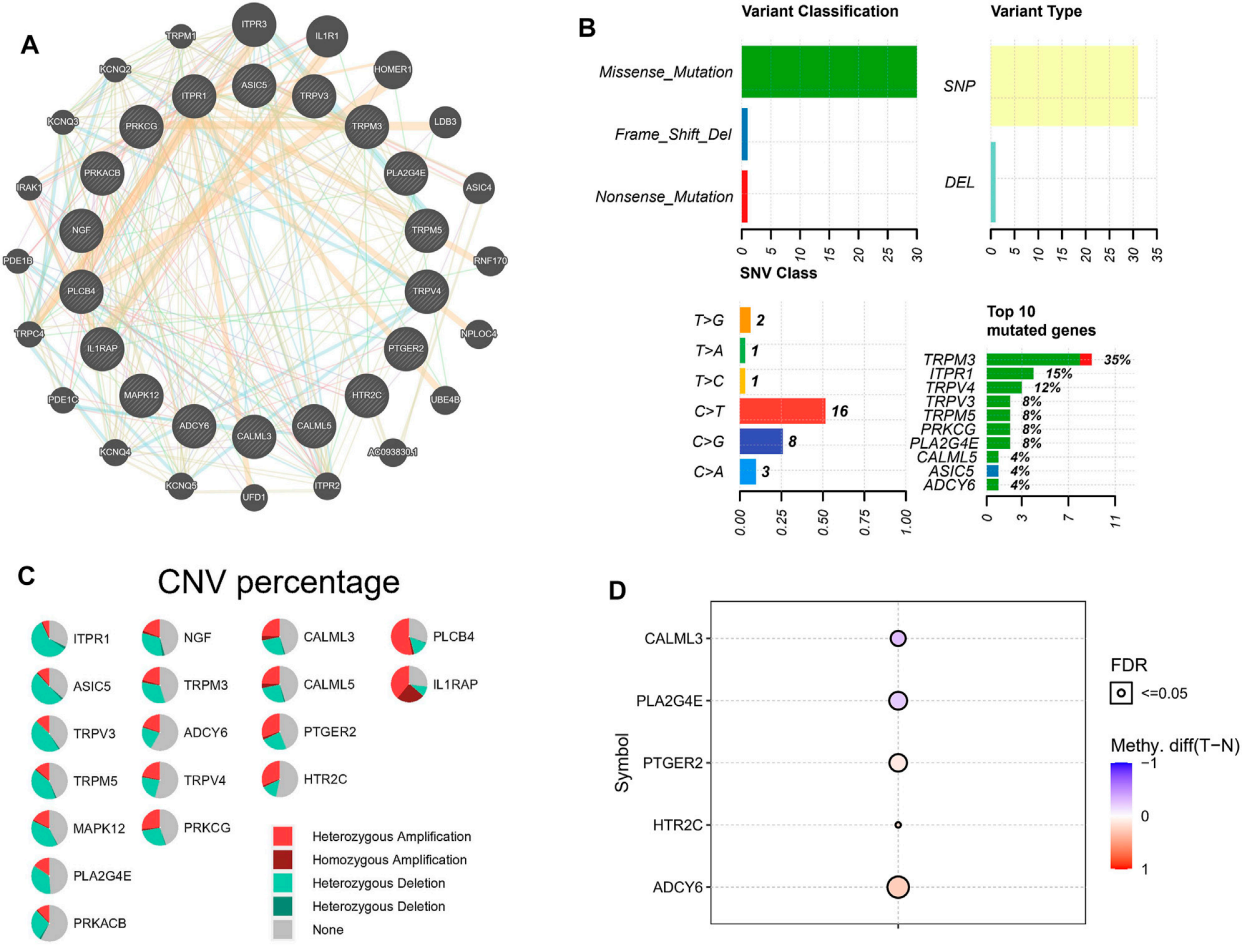
TRP channel-related genes mutational landscape. **(A)** A PPI network of 18 TRP channel-related DEGs. **(B)** The mutation of 18 TRP channel-related DEGs. **(C)** The CNV variation of 18 TRP channel-related DEGs. **(D)** Differences in the degree of methylation of PTGER2, HTR2C, ADCY6, CALML3, and PLA2G4E in normal and tumor samples.

To investigate the interaction between 18 TRP channel-associated DEGs, the online tool of the GeneMANIA website was employed to build the PPI network. Relatively close interactions were observed among the 18 TRP-channel-related DEGs (Figure 3A). We downloaded mutation data from TCGA database and analyzed 18 TRP channel-related DEGs. As shown in Figure 3B, missense mutations were the most common variant. Further, SNPs were the most common variant kind, and C > T was ranked as the top SNV class. Notably, TRPM3 had the highest mutation frequency among the 18 TRP channel-related DEGs.

We also revealed the CNV status of 18 TRP channel-related DEGs in ESCC patients. The results showed that most genes had heterozygous amplification and deletions (Figure 3C). Normal samples were used as controls to study the methylation of these genes. Interestingly, among the 18 TRP channel-related DEGs, only CALML3, PLA2G4E, PTGER2, HTR2C, and ADCY6 showed obvious diversities in the degree of methylation between normal samples and ESCC samples. Specifically, the degree of methylation of PTGER2, HTR2C, and ADCY6 was higher than that in normal samples; the degree of methylation of CALML3 and PLA2G4E was lower than that in normal samples (Figure 3D). Unfortunately, only the methylation degreels of above five TRP channel-related DEGs can be retrieved from the TCGA database. Finally, we estimated the role of each gene in tumor-related pathways using the GSVA algorithm, including apoptosis, DNA damage response, cell cycle, hormone AR, hormone ER, PI3K/AKT, EMT, RAS/MAPK, RTK, and TSC/mTOR. Based on the results, TRP channel-related genes can activate most of the above pathways, while inhibition only includes a few pathways, such as apoptosis and the cell cycle (Supplementary Figure S2).

### Development of a Prognostic Signature in the GEO Cohort

**FIGURE 4:**
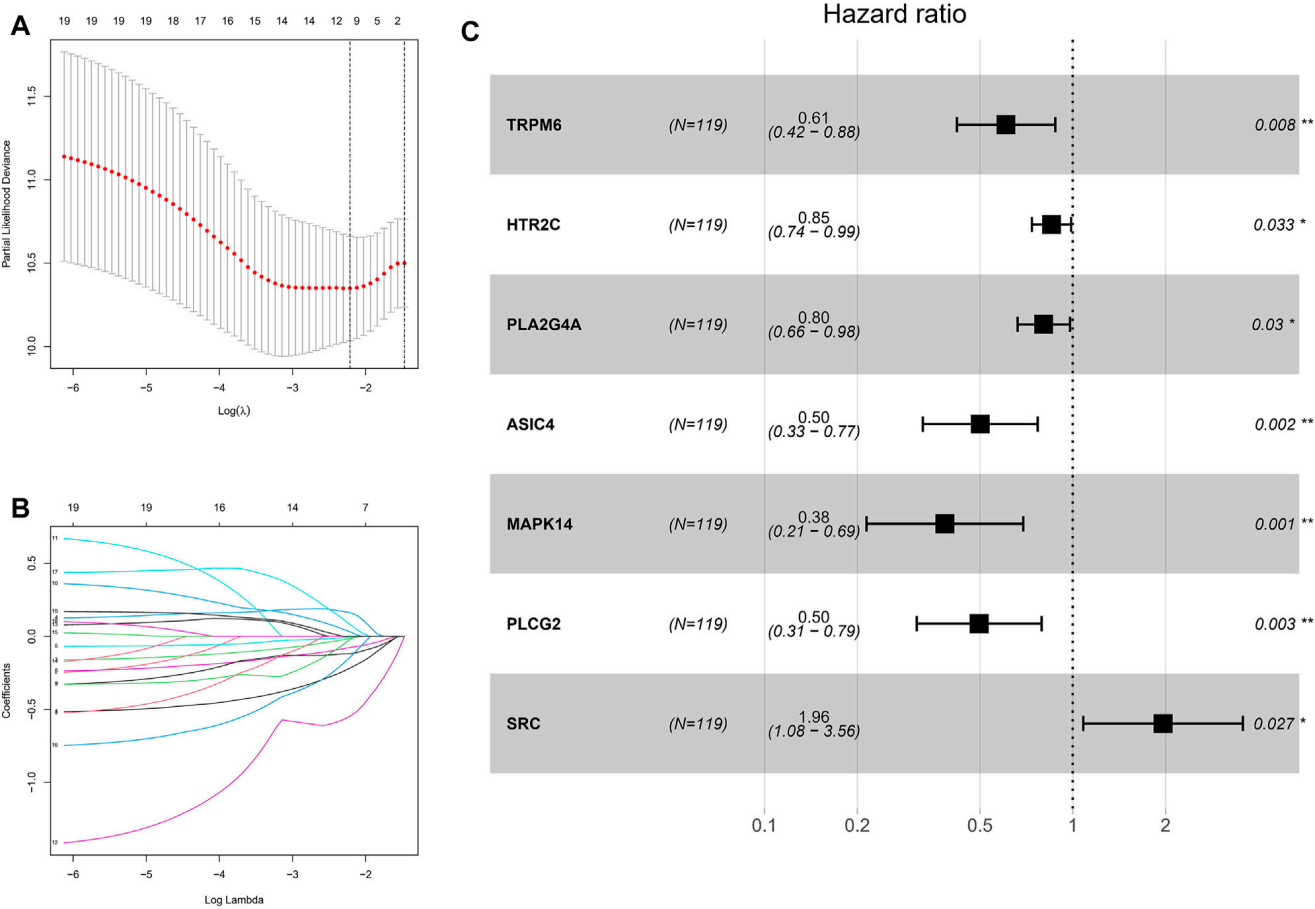
Construction of risk signature in the GEO cohort. **(A)** Cross-validation for tuning the coefficient selection in the LASSO regression. **(B)** LASSO regression of the 19 OS-associated genes. **(C)** Multivariate Cox regression analysis for 7 genes.

Not only are ion channels dysregulated in cancer, but the expression of their regulators, effectors and other interacting genes is also significantly altered. TRP channels, as a typical ion channel, we speculate that the altered gene expression of related channels would reflect the risk of tumor progression from another perspective. To calculate TRP scores in ESCC patients, we screened 19 genes associated with prognosis in the GEO-ESCC cohort by univariate Cox regression analysis (*p* < 0.1), and further screened by LASSO regression analysis. The prognostic signature performed best when seven genes were included (Figures 4A,B). Finally, the regression coefficients of the seven genes were calculated using multivariate Cox regression analysis (Figure 4C). The signature formula is: TRP risk score = (−0.498711399 × expression level of TRPM6) + (−0.15894075 × expression level of HTR2C) + (−0.217314244 × expression level of PLA2G4A) + (−0.690911672 × expression level of ASIC4) + (−0.949344105 × expression level of MAPK14) + (−0.699670637 × expression level of PLCG2) + (0.673798617 × expression level of SRC).

### Prognosis Prediction Based on Transient Receptor Potential Risk Score

**FIGURE 5:**
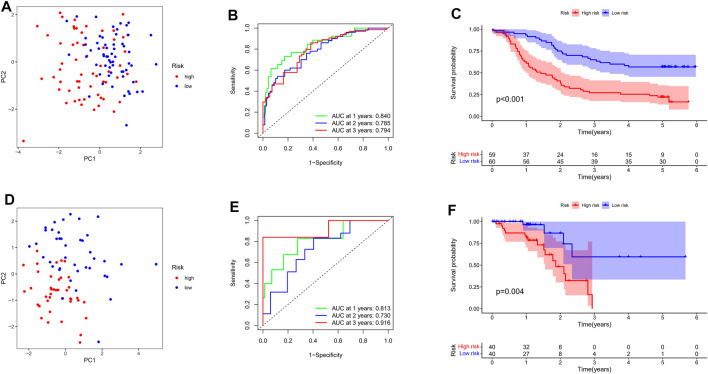
Evaluation the prognostic value of TRP channel-related genes signature. **(A)** Allocation of patients in the training set on basis of the risk score. **(B)** AUC of time-dependent ROC curves examined the prognostic performance of the risk score in the training set. **(C)** Kaplan-Meier curves display the diversity in OS between the high-risk and low-risk groups in the training set. **(D)** Allocation of patients in the verification set on basis of the risk score. **(E)** AUC of time-dependent ROC curves examined the prognostic performance of the risk score in the verification set. **(F)** Kaplan-Meier curves display the diversity in OS between the high-risk and low-risk groups in the verification set.

Eighty patients with TGCA-ESCC were used as an external validation cohort. The gene expression data were standardised using the “sva” package before further analysis (Supplementary Figure S3). Based on the median value of risk scores, 80 patients in the TCGA cohort were divided into the high-risk group (*n* = 40) and the low-risk group (*n* = 40), and 119 patients in the GEO cohort were divided into the high-risk group (*n* = 59) and the low-risk group (*n* = 60). PCA analysis showed that both risk subgroups in both cohorts were well separated (Figures 5A,D). ROC analysis of the GEO-ESCC cohort showed a good predictive effect of the risk score (1-year AUC = 0.840, 2-year AUC = 0.785, and 3-year AUC = 0.794) (Figure 5B). Similarly, good predictive power was shown in the TGCA-ESCC cohort (1-year AUC = 0.813, 2-year AUC = 0.730, and 3-year AUC = 0.916) (Figure 5E). The Kaplan-Meier curve consistently showed that the OS of the high-risk subgroup was substantially shorter than that of the low-risk subgroup (*p* < 0.05, Figures 5C,F).

### Independent Prognostic Value of Transient Receptor Potential Risk Score

**FIGURE 6:**
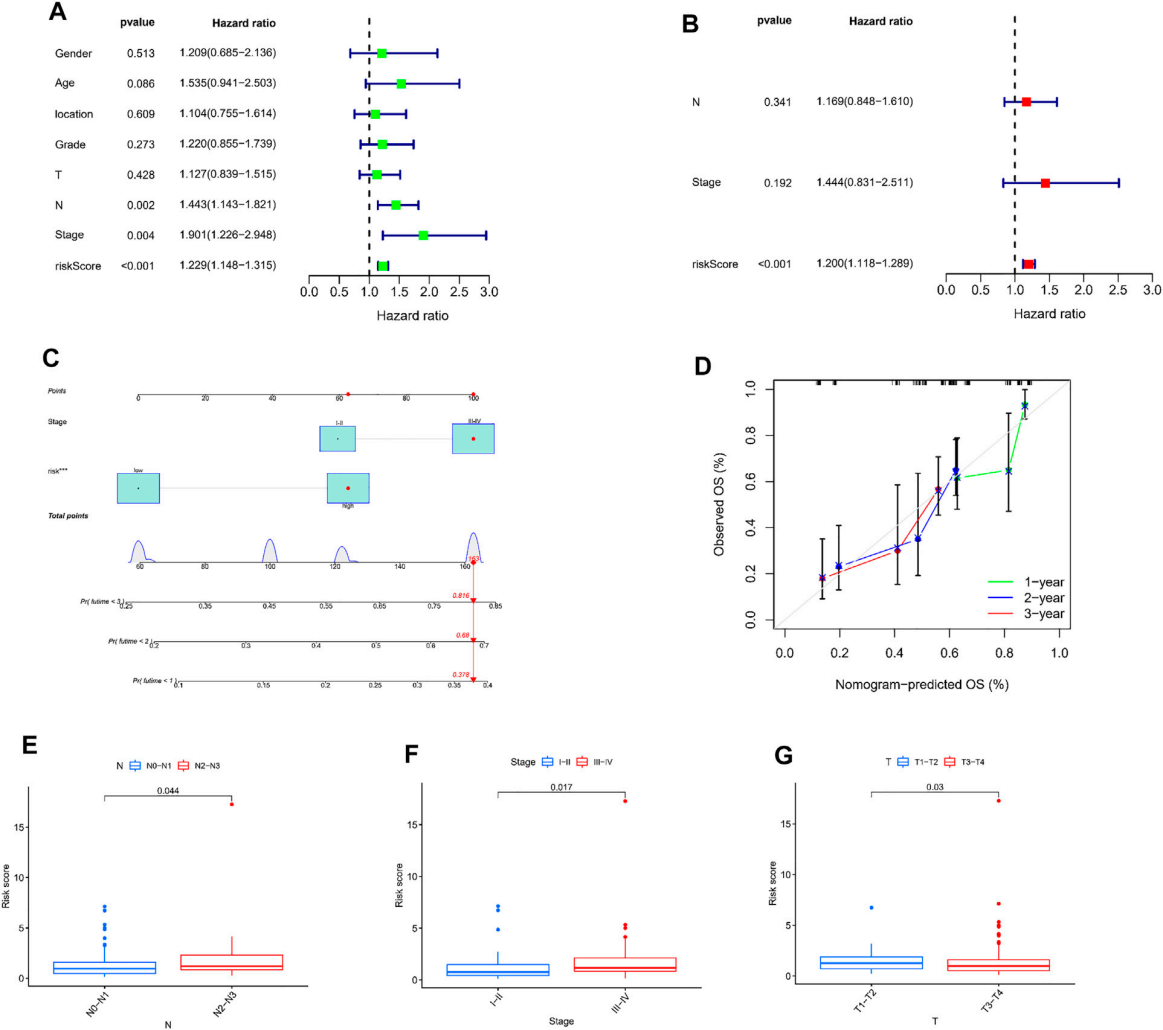
Independent prognostic value of TRP risk score. **(A,B)** Univariate and multivariate Cox regression analysis of the risk scores. **(C)** A nomogram on basis of TNM phase and risk score. **(D)** Calibration curves of 1, 2, and 3 year. The association between risk score and N stage **(E)**, TNM stage **(F)** and T stage **(G)**.

Combined with the clinical data, we again performed univariate and multivariate Cox regression analysis to assess whether the TRP risk score could be used as an independent predictor for ESCC. Univariate Cox regression analyses revealed that the risk score (*p* < 0.001, HR = 1.229), N stage (*p* = 0.002, HR = 1.443), and TNM stage (*p* = 0.004, HR = 1.901) were obviously associated with OS (Figure 6A). Multifactorial Cox regression analysis showed that only risk score was an independent predictor for OS (*p* < 0.001, HR = 1.200, Figure 6B).

Considering the importance of TNM staging in clinical practice, we combined TNM staging with TRP risk scores to construct a nomogram to predict 1-, 2-, and 3-year survival (Figure 6C). Besides, the calibration curve for the probability of 1, 2, and 3-year OS showed an optimal agreement between observation and prediction (Figure 6D). Moreover, TRP risk score with N stage (*p* = 0.044, Figure 6E), TNM stage (*p* = 0.017, Figure 6F), and T stage (*p* = 0.03, Figure 6G) showed significant differences.

These results suggest that TRP risk scores may influence the clinical outcomes of ESCC patients.

### Comprehensive Immune Analysis

**FIGURE 7:**
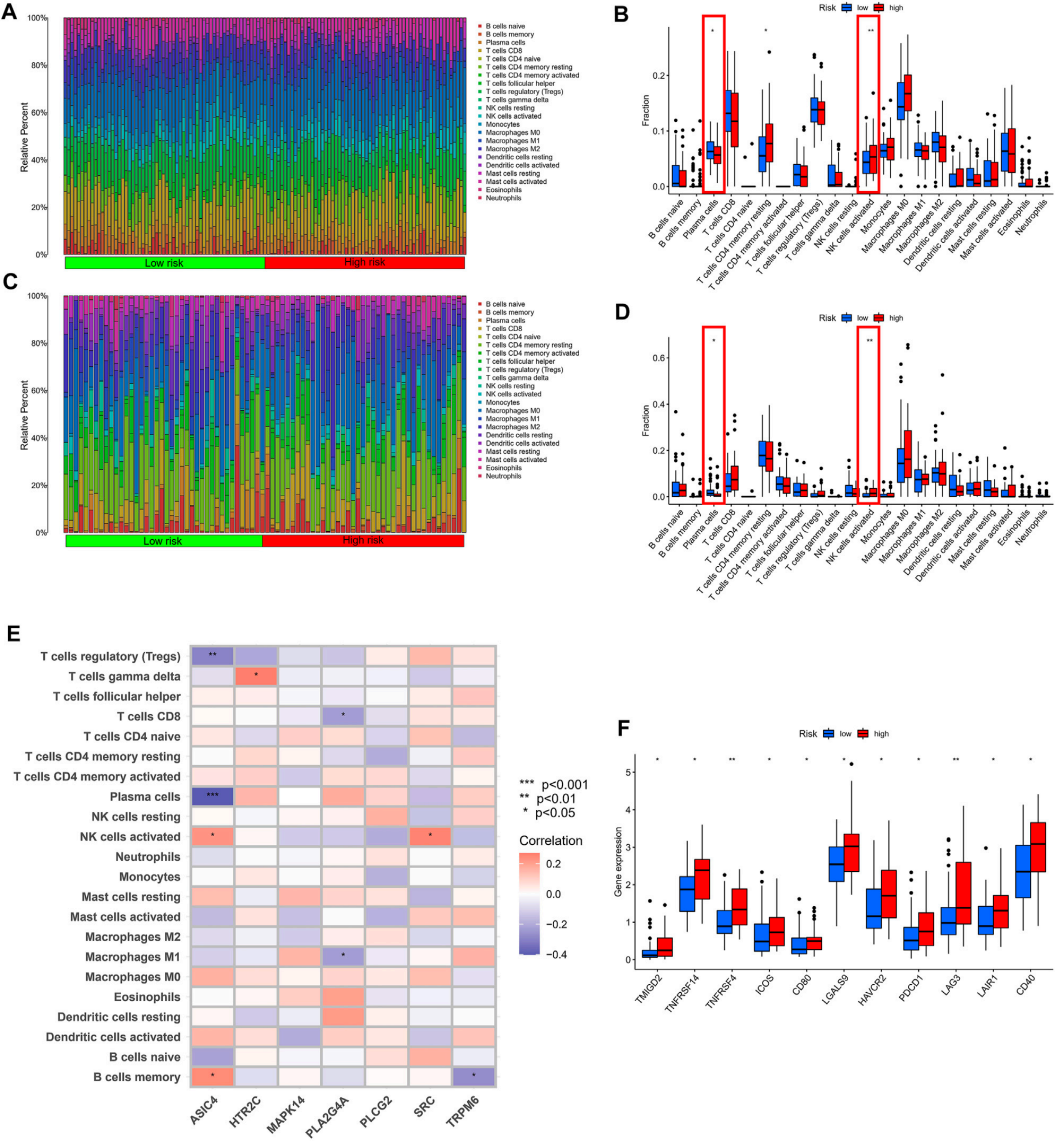
Diversities in immune microenvironment between high- and low-risk groups. **(A,C)** Immune cell kind percents in the low- and high-risk groups. **(B,D)** Differential immune infiltrates in the high-risk and low-risk groups. **(E)** Association between 7-gene signature expression and the infiltration level of immune cells. **(F)** Expression levels of immune checkpoint proteins in high-risk and low-risk ESCC patients. *p < 0.05, ***p < 0.001, ****p < 0.0001, ns, not significant.

We further comprehensively evaluated the guiding role of TRP risk score in immunotherapy and immune-related mechanisms. As shown in Figures 7A,C, macrophages, such as those belonging to the M0, M1, and M2 subsets, constituted a significant proportion in both TCGA and GEO cohorts. The distribution of immune cell subsets between the low- and high-risk groups was shown using a box plot. In the low-risk group of the TCGA and GEO cohorts, the infiltrating scores of plasma cells was obviously higher and NK cells activated lower (Figures 7B,D). Meanwhile, in the GEO cohort, Pearson analysis was used to explore the relationship between seven signature-related genes and the level of 22 immune cell infiltrations. We found that ASIC4 was positively correlated with B cells memory, NK cells activated and negatively correlated with plasma cells. HTR2C was only positively correlated with T cells gamma delta. PLA2G4A was negatively correlated with macrophages M0, T cells CD8. Additional details were shown in Figure 7E. In addition, Figure 7F showed only the immune checkpoints with differential expression (TMIGD2, TNFRSF14, TNFRSF4, ICOS, CD80, LGALS9, HAVCR2, PDCD1, LAG3, LAIR1, CD40), all of which were upregulated in the high-risk group compared to the low-risk group.

The tumor purity score, estimate score, immune score, and stromal score were successfully generated with the ESTIMATE algorithm. Remarkably, patients who had a low-risk score demonstrated greater tumor purity (*p* = 0.038), lower estimate score (*p* = 0.038), lower immune score (*p* = 0.044), and higher stromal score (*p* = 0.045) than those who had a high-risk score (Supplementary Figures S4A–D), which was consistent with previous research findings that a lower estimate score indicates greater tumor purity. Human leukocyte antigen (HLA)-related genes exert a vital effect on controlling the immune response. After comparing the expression of HLA-associated genes among various groups, it was observed that most of the HLA-associated genes are upregulated in the high-risk group (Supplementary Figure S4E).

Additionally, the correlation between 7 signature-associated genes and immune infiltration in the ESCC microenvironment was established in order to get a better understanding of the relationship between these genes and immune infiltration. According to the findings, the expression of the majority of genes was shown to be negatively related to the level of immune cell infiltration (Supplementary Figure S5). Finally, we analyzed the transcriptional regulatory network of seven signature-associated genes, in which MAPK14 regulated the most TF and dominated the transcriptional regulatory network (Supplementary Figure S6).

### Gene Set Enrichment Analyses Analysis

**FIGURE 8:**
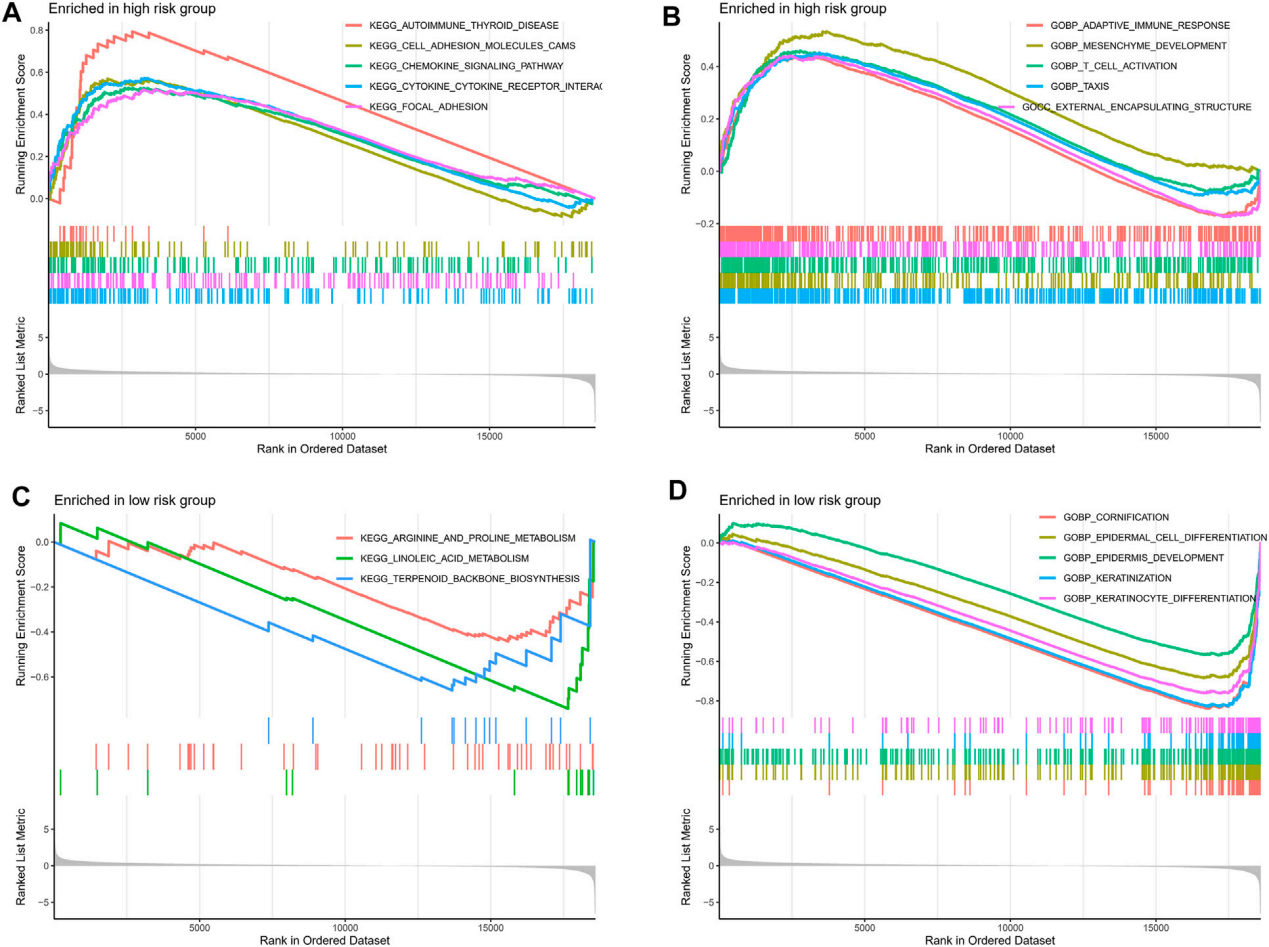
The GSEA analysis of the high- and low-risk groups. The KEGG pathway enrichment analysis of the high- **(A)** and low-risk **(C)** groups. The GO pathway enrichment analysis of the high- **(B)** and low-risk **(D)** groups.

To further explore the potential mechanisms underlying the different clinical risks caused by abnormal TRP-related gene expression, we performed a GSEA analysis. The outcomes revealed that immune-associated pathways such as chemokine signaling pathway, cytokine-cytokine receptor interaction, adaptive immune response, and T cell activation were enhanced in the high-risk group (Figures 8A,B). Interestingly, the significant enrichment of metabolism- and cell growth-associated pathways was observed in the low-risk group, including arginine and proline metabolism, linoleic acid metabolism, terpenoid backbone biosynthesis, and keratinocyte differentiation (Figures 8C,D).

### Drug Sensitivity Analysis

**FIGURE 9:**
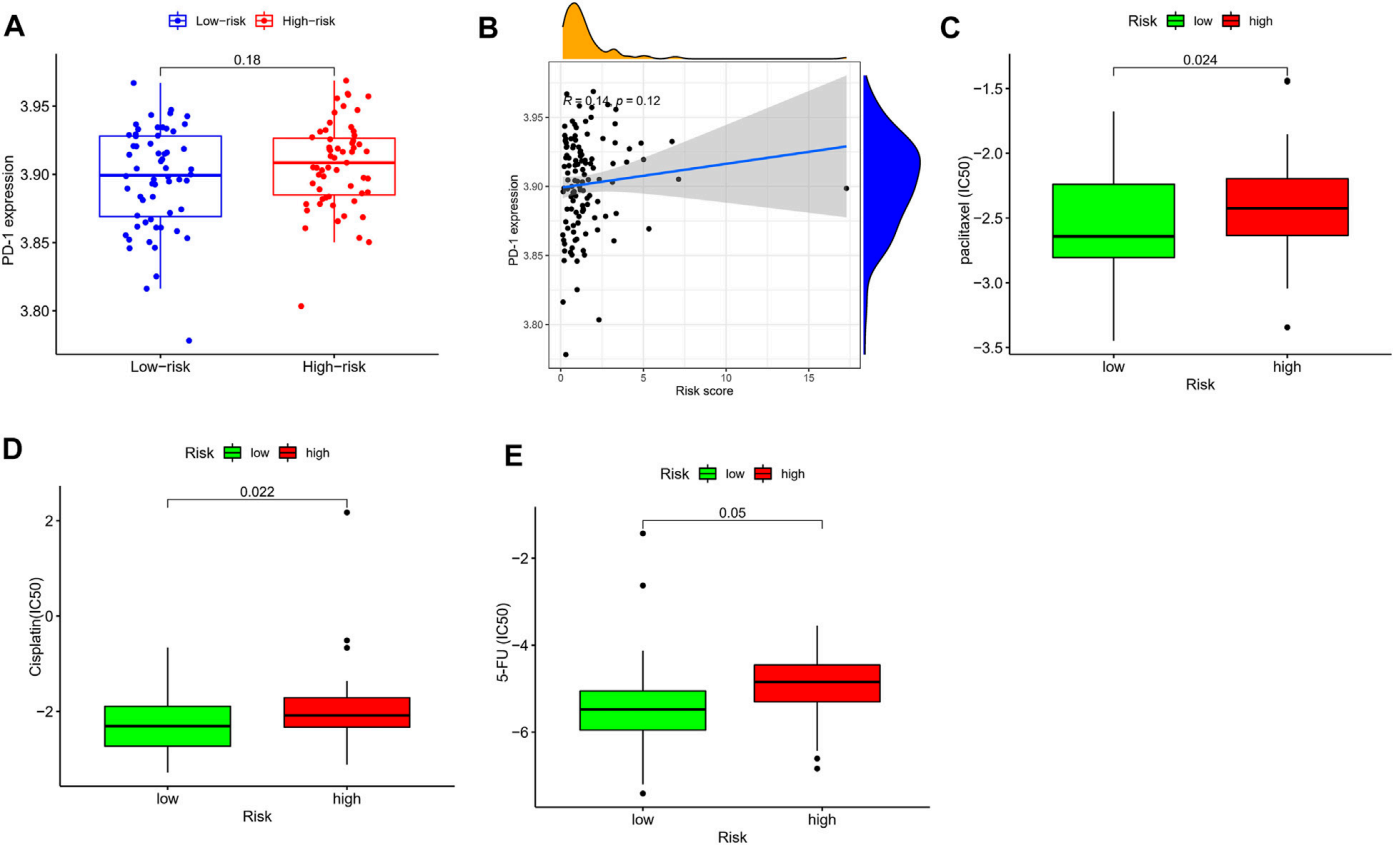
Drug sensitivity analysis. **(A)** The expression level of PD-1 in different risk groups. **(B)** Association of the risk score with the PD-1 expression in ESCC samples. The IC50 values of three chemo drugs in the low- and high-risk groups including paclitaxel **(C)**, cisplatin **(D)**, and 5-FU **(E)**.

The current first-line chemotherapy regimens for ESCC in clinical guidelines include platinum-containing dual-agent chemotherapy regimens, paclitaxel combined with platinum regimens, or 5-fluorouracil-based regimens. In addition, anti-PD-1 drugs also have an important role in ESCC. We used the pRRophetic algorithm to evaluate the therapeutic effect of drugs. Unfortunately, there was no difference in PD-1 expression between different risk groups (Figure 9A), and correlation analysis also showed a weak correlation (Figure 9B). This reminded us that the novel immune checkpoint identified in Figure 7F might be useful for the treatment of patients with different TRP risks. In addition, the IC50 results were encouraging: the low-risk group showed better efficacy for paclitaxel (Figure 9C), cisplatin (Figure 9D), and 5-fluorouracil (Figure 9E) than the high-risk group, indicating that the TRP risk score plays a guiding role in the chemotherapy of ESCC patients.

### External Validation of 7 Signature-Associated Genes

**FIGURE 10:**
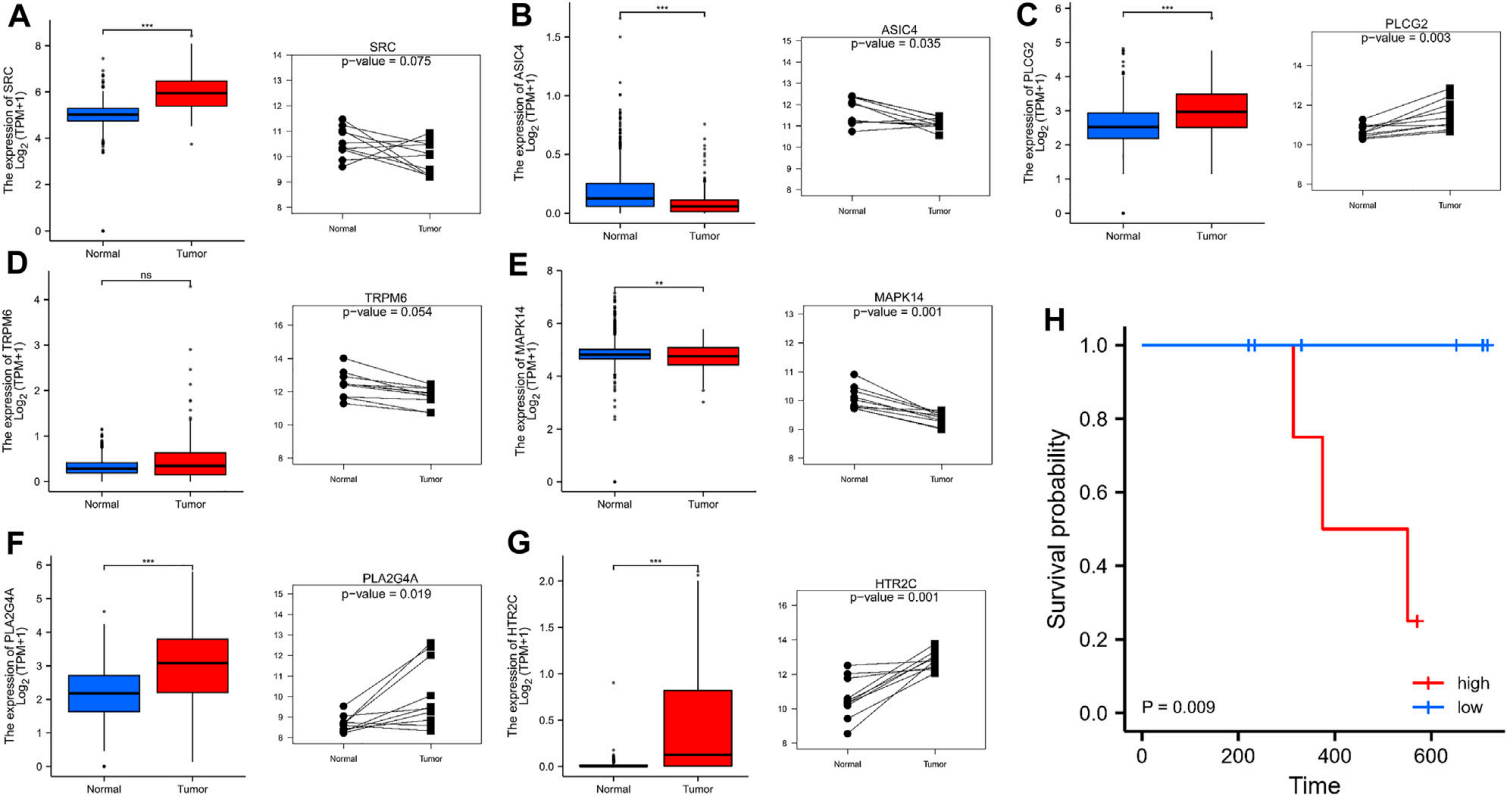
**(A–G)** The expression of 7 signature-associated genes between tumor tissues and normal esophageal tissues on basis of the GTEx database and qRT-PCR. **(H)** qRT-PCR of the expression of 7 signature-associated genes in 10 pairs tissues.

We used the GTEx database and qRT-PCR experiment to analyze the expression levels of the 7 signature-associated genes were analyzed with the GTEx database and qRT-PCR experiment. As shown in Figures 10A–G, tumor tissues showed obviously higher expression levels of PLCG2, PLA2G4A, and HTR2C than the normal esophageal tissues. There was high expression of ASIC4 and MAPK14 in normal esophageal samples. Nevertheless, no obvious diversity was observed in the expression level of TRPM6 between ESCC and nearby tissues. The above-mentioned gene expression results in clinical tissue samples almost conformed to the RNA sequencing data analyzed by the TCGA database. In addition, results also showed that high-risk cohort had a shorter survival time in our hospital cohort (*p* < 0.05). Survival analysis also showed that high-risk cohort had a shorter survival time in our hospital cohort (*p* < 0.05) (Figures 10H).

## Discussion

The purpose of this work was to investigate the expression patterns of TRP channel-related genes in ESCC, as well as their prognostic significance and function in the TME. The expression of TRPM5, TRPM3, PLCB4, ITPR1, PTGER2, ADCY6, PRKACB, and PRKCG was obviously declined in ESCC tissues by comparing with that in normal tissues, whereas TRPV4, TRPV3, HTR2C, PLA2G4E, ASIC5, CALML3, CALML5, IL1RAP, MAPK12, and NGF expression were significantly increased. Consensus clustering was Adopted to determine two subtypes of ESCC, cluster 1 and cluster 2 on basis of the expression profiles of 18 TRP channel-related DEGs. It has been noted that patients in cluster 1 are closely associated with advanced tumor stage and grade. According to the prediction, cluster 2 showed a better survival probability than cluster 1. A prognostic prediction signature was constructed for the GEO-ESCC cohort. The risk scoring system, which was developed on the basis of 7 genes, was used to predict the prognosis of ESCC patients. Further, the effective stratification of patients into high- and low-risk groups was conducted. Patients in the high-risk group showed an obviously lower survival probability than those in the low-risk group. The TCGA-ESCC cohort confirmed the performance of the prognostic signature. Besides, the risk score increased as the tumor progressed and became severe. Cox analysis of univariate and multivariate revealed that the 7-gene signature was an independent factor.

Among the 7 signature-associated genes, PLCG2 has been involved in regulating cell proliferation, transformation, and tumor growth (Ma et al., 2019). TRPM6 is reported to be downregulated in colorectal cancer, with high expression related to better patient survival (Xie et al., 2018). Bing et al. (2018) suggested that HTR2C plays a role in the non-small-cell lung cancer pathway, directly influencing epidermal development element receptor tyrosine kinase inhibitor resistance. PLA2G4A acts as an important enzyme involved in tumor development. In addition to upregulating the level of E-cadherin, PLA2G4A downregulated the level of vimentin, curbing ESCC cell mobility and invasion (Zhao et al., 2018). It has been considered that SRC is notoriously morbific for inducing carcinogenesis in the methods of proliferation, adhesion, angiogenesis, invasion, and apoptosis (Luo et al., 2009). According to accumulating evidence, oncogenic SRC is in charge of tumor progression and participates in resisting against anticancer drugs in traditional and targeted therapies (Adams et al., 2016). One previous research found that 80% of patients with colon cancer overexpress SRC in tumor tissue, and while accelerating metastasis, the overexpression of SRC in colon cancer results in chemotherapeutic drug resistance *via* various downstream signaling pathways (Chen et al., 2014). According to these studies, dysregulation of TRP channel-associated genes might exert divergent effects in various kinds of cancer.

The TME has a vital regulatory effect on carcinogenesis and tumor progression (Lin et al., 2016). TRP channels may have an impact on the TME by regulating the interactions between the sensory-vascular-immune-tumor systems (Khalil et al., 2018; Saldías et al., 2021). Based on our scoring system, there was notable diversity in the TME between the low-risk and high-risk groups. The immune scores and expression levels of HLA-related genes in the high-risk group were considerably greater than those in the low-risk group, although tumor purity exhibited the reverse tendency, which may explain the higher survival of patients in the low-risk group. Our findings aligned with those reported by Zeng et al. (2018), indicating that patients with low immune scores show better OS than patients with high immune scores. ESCC is regarded as an immunogenic tumor. Nevertheless, to a great extent, immune dysfunction is mediated by inducing immunosuppressive cells to infiltrate the TME (Huang and Fu, 2019). Currently, investigation on the TRP channel-related genes in the TME in ESCC is inadequate. According to our research, the expression of most signature-related genes was negatively related to the infiltrating levels of immune cells. These outcomes show that the TRP channel partially regulates the TME. In addition, to further explore the potential mechanisms of different clinical risks caused by abnormal expression of TRP channel-related genes, the related biological processes and signaling pathways associated with the high-and low-risk groups were investigated via GSEA. The findings revealed that immune-associated pathways, such as the chemokine signaling pathway, cytokine-cytokine receptor interaction, adaptive immunological response, and T cell activation, were enriched in the high-risk group than in the control group. In conclusion, the findings indicate that the prognostic signature may serve as an indication of immune cell infiltration, which has significant implications for clinical practice.

Many clinical studies are now being conducted to assess the function of immune checkpoint inhibitors in the treatment of patients with ESCC. By investigating the relationship between the risk score and the expression of essential immune checkpoints, it was discovered that the majority of immune checkpoints were expressed at greater levels in the high-risk group. On basis of these findings, the vital effect of the immunosuppressive microenvironment on patients with a poor prognosis is suggested. Therefore, patients with high risk scores might better benefit from immunotherapy than patients with low risk scores. Based on drug sensitivity analysis, it was likely that patients in the low-risk group respond well to chemotherapy drugs. These outcome displays that TRP channel-associated genes may forecast or impact the therapeutic roles in patients with ESCC.

Certainly, the potential limitations of the study should be noted. First, the sample size and training cohort are relatively small for the estimation of a predictive model, so the results of the model are only a suggestion for future developments. Second, we should collect the clinical data of our medical center for further validation of the nomograms to make the results more reliable. Third, although we confirmed the expression in our own samples through qRT-PCR, larger sample size should also be used to provide further validation.

In summary, the prognostic value, roles in the TME, response to immune checkpoints, and drug sensitivity of TRP channel-associated genes in ESCC were analyzed in a systematic manner. ESCC patients can be stratified into low- and high-risk subgroups with various prognoses based on the prognostic signature generated by 7 TRP channel-related genes. According to the signature, patients with high-risk scores might benefit most from immunotherapy, and patients with low risk scores were more sensitive to chemotherapy drugs. Finally, GTEx database and qRT-PCR outcomes had determined the differential expressions of signature-associated genes in the tissue samples. Our research provides a new gene signature for forecasting the prognosis of ESCC patients and a significant basis for future research on the associations between TRP channels and TME in ESCC.

## Data Availability

The original contributions presented in the study are included in the article/Supplementary Material, further inquiries can be directed to the corresponding author.

## Supplementary Material

Supplementary Figure S1 Flowchart of the present study.

Supplementary Figure S2 Distribution of 18 TRP channel-related DEGs in tumor related pathways.

Supplementary Figure S3 Batch results of GEO and TCGA cohorts. **(A)** PCA analysis before batch. **(B)** PCA analysis after batch.

Supplementary Figure S4 Different risk groups in ssGSEA and Estimate algorithms display different immune statuses. Expression level of the tumor purity **(A)**, ESTIMATE score **(B)**, immune score **(C)**, stromal score **(D)**, and HLA-associated genes **(E)** between the low- and high-risk groups. **p* < 0.05, ****p* < 0.001, *****p* < 0.0001, ns, not significant.

Supplementary Figure S5 Correlation of 7 signature-related genes expressions with immune infiltration levels in ESCC. **(A)** TRPM6, **(B)** HTR2C, **(C)** PLA2G4A, **(D)** MAPK14, **(E)** PLCG2, **(F)** SRC, and **(G)** ASIC4.

Supplementary Figure S6 Regulatory network analysis.
