# Supplementary figures and images for: Comprehensive Analysis of TRP Channel-Related Genes for Estimating the Immune Microenvironment, Prognosis, and Therapeutic Effect in Patients With Esophageal Squamous Cell Carcinoma
Source: Front Cell Dev Biol. 2022 Mar 4;10:820870. doi: 10.3389/fcell.2022.820870 (PMC8931688; doi:10.3389/fcell.2022.820870)

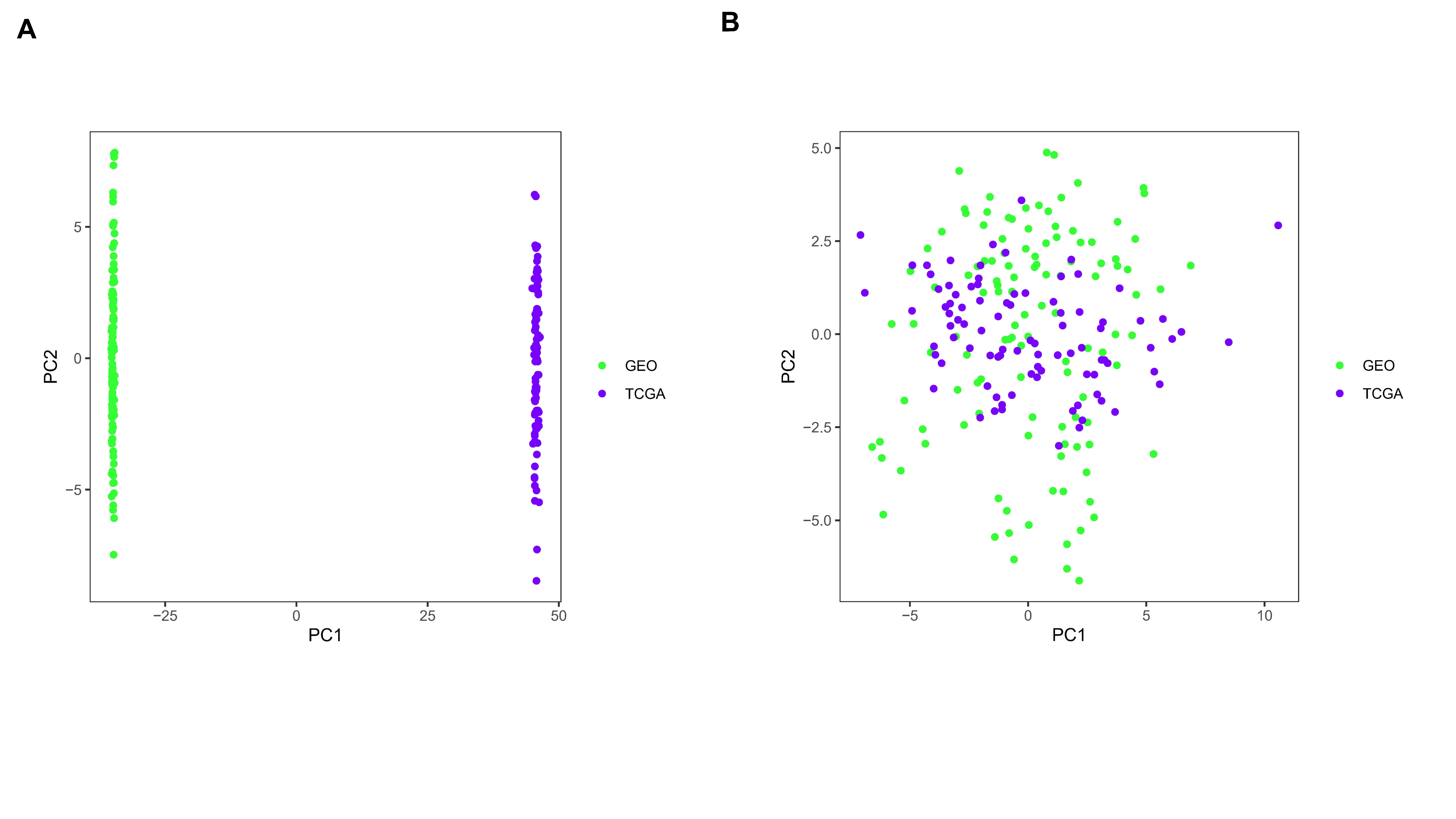

Supplement: Supplementary file 1 [file Image3.TIFF]

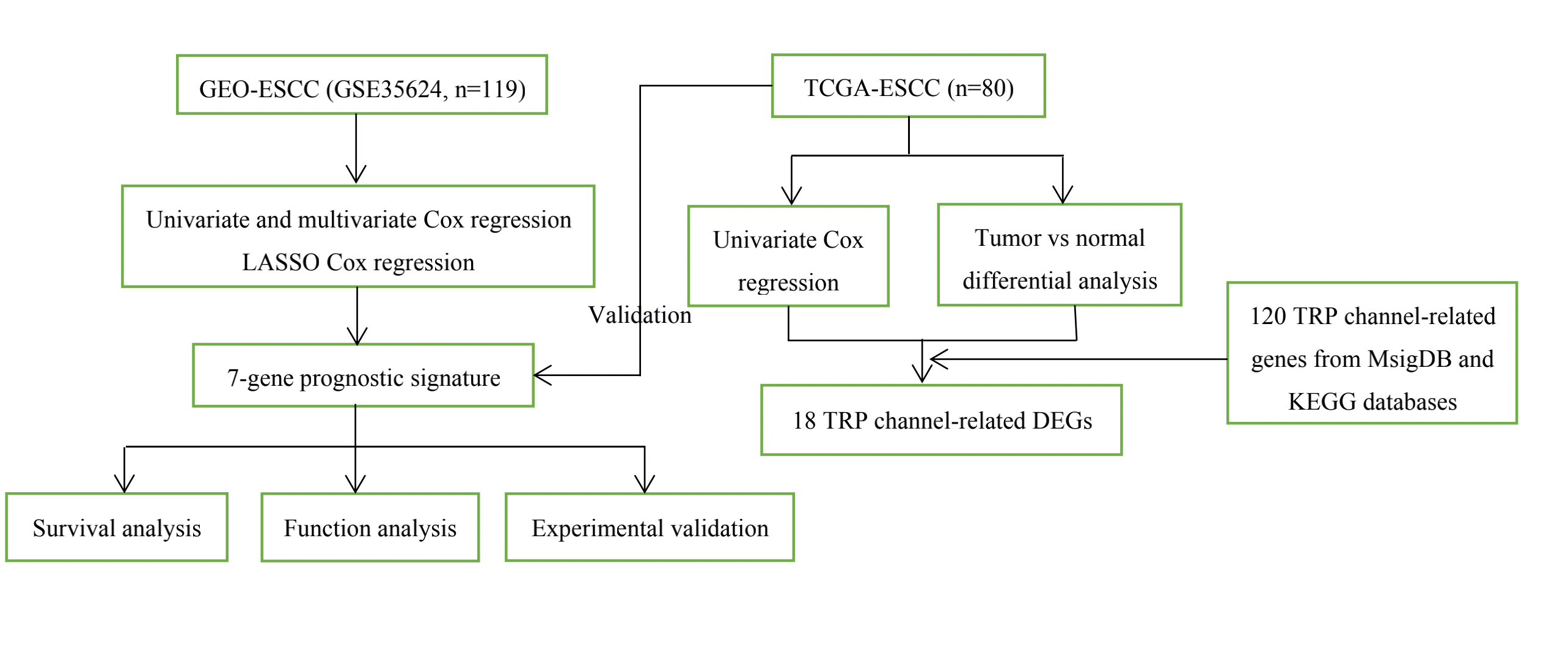

Supplement: Supplementary file 2 [file Image1.TIFF]

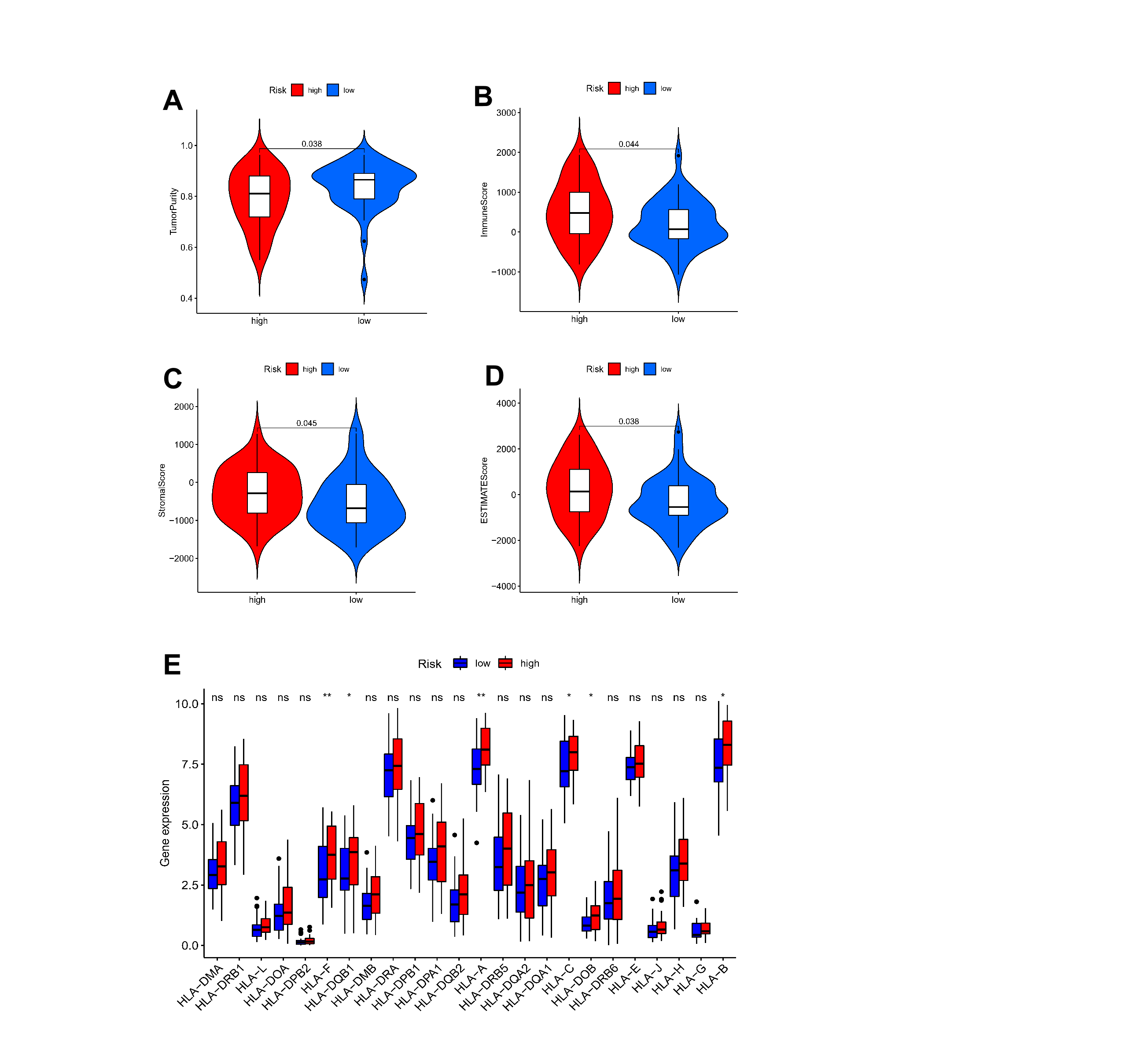

Supplement: Supplementary file 4 [file Image4.TIF]

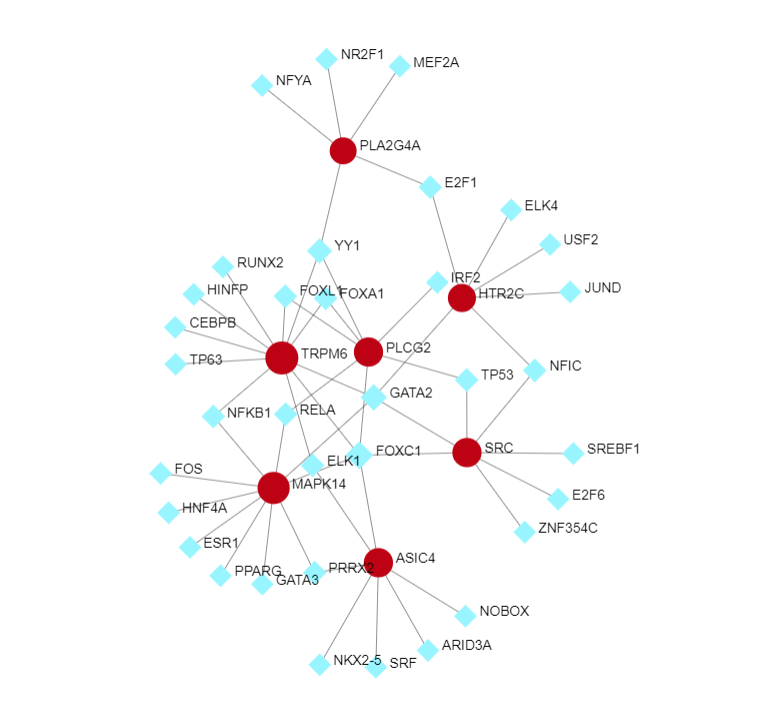

Supplement: Supplementary file 5 [file Image6.TIFF]

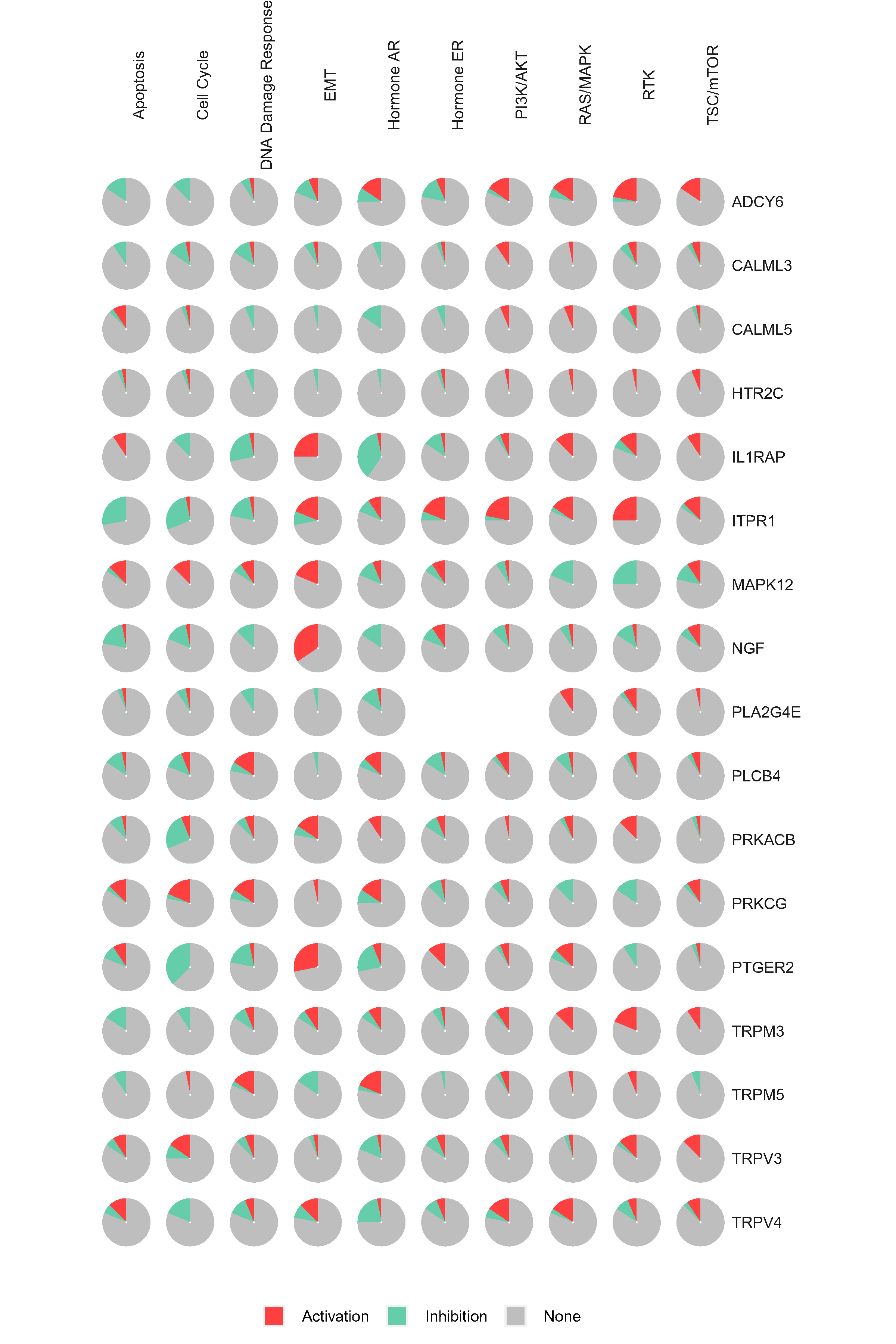

Supplement: Supplementary file 6 [file Image2.TIFF]
